# Coronavirus disease 2019 (COVID-19) era hospital infection controls reduce other serious infections and must be continued after the COVID-19 tragedy is resolved

**DOI:** 10.1017/ice.2021.11

**Published:** 2021-01-25

**Authors:** Luke Curtis

**Affiliations:** Curtis Research, Hazelwood, Missouri

*To the Editor—*I read with great interest your 3 recent papers by Wong et al^1^ Ponce-Alonso et al^2^, and Wee et al,^3^ who report that coronavirus disease 2019 (COVID-19)–era infection control bundles are associated in significant decreases in many nosocomial viral and bacterial infection rates such as influenza, respiratory syncytial virus, adenovirus, and *Clostridium difficile*. The Ponce-Alonso Spanish study reported a decrease of 69% in hospital-acquired *Clostridium difficile* infection rates following implementation of a hospital infection control bundle: incidence density was 8.54 per 10,000 patients day before and 2.68 per 100,000 days after the COVID-19 infection control bundle (*P* = .000257^2^).

Other studies have also reported that COVID-19–era infection controls such as hand washing, masking and gowning, better hospital cleaning, and isolation of COVID-19 patients can significantly reduce rates of many bacterial and viral infections. A study in a 1,785-bed Singapore hospital reported that the use of COVID-19–related infection control bundles was associated with significantly reduced rates of many hospital-acquired infections, including nosocomial respiratory infections (incidence rate, 0.08; 95% CI, 0.05–0.13), nosocomial MRSA (IR, 0.54; 95% CI, 0.46–0.64), and central-line bloodstream infections (IR, 0.24; 95% CI, 0.07–0.57).^4^ A California study involving 37,033 hospital patient days reported that following implementation of a COVID-19 infection control bundle, rates of many multidrug-resistant pathogens decreased significantly including a 41% decrease in methicillin-resistant *Staphylococcus aureus* (MRSA), a 21% decrease in (extended-spectrum β-lactamase bacteria (ESBL), and an 80% decrease in vancomycin-resistant enterococci (VRE).^5^

Better hospital infection control bundles can also reduce rates of common nosocomial fungal infections,^6^ although I am not aware of any current studies that have reported on lower rates of *Aspergillus* or *Candida* in the COVID-19 era. Such studies of COVID-19–era nosocomial *Aspergillus* and *Candida* rates might yield useful data on how to prevent common life-threatening fungal infections.

Clearly, enhanced infection control procedures need to be followed long after the COVID-19 tragedy has resolved.^7^ I hope that *Infection Control and Hospital Epidemiology* will continue to publish more good papers on COVID-19–era infection control.

## References

[1] Wong SC , Lam GK , AuYeung CH , et al. Absence of nosocomial influenza and respiratory syncytial virus infection in the coronavirus disease 2019 (COVID-19) era: Implication of universal masking in hospitals. Infect Control Hosp Epidemiol 2020. doi: 10.1017/ice.2020.425. PMC7468684 32799965

[2] Ponce-Alonso M , Sáez de la Fuente J , Rincón-Carlavilla A , et al. Impact of the coronavirus disease 2019 (COVID-19) pandemic on nosocomial *Clostridioides difficile* infection. *Infect Control Hosp Epidemiol* 2020. doi: 10.1017/ice.2020.454. PMC7520631 32895065

[3] Wee LE , Conceicao EP , Sim JXY , Aung MK , Venkatachalam I. Impact of infection prevention precautions on adenoviral infections during the COVID-19 pandemic: experience of a tertiary hospital in Singapore. *Infect Control Hosp Epidemiol* 2020. doi: 10.1017/ice.2020.1365. PMC8770837 33298224

[4] Wee LEI , Conceicao EP , Tan JY , et al. Unintended consequences of infection prevention and control measures during COVID-19 pandemic. *Am J Infect Control* 2020. doi: 10.1016/j.ajic.2020.10.019. PMC7610096 33157180

[5] Cole J , Barnard E. The impact of the COVID-19 pandemic on healthcare acquired infections with multidrug-resistant organisms. *Am J Infect Control* 2020:5726. doi: 10.1016/j.ajic.2020.09.013. PMC7529600 33011335

[6] Suleyman G , Alangaden GJ. Nosocomial fungal infections: epidemiology, infection control, and prevention. Infect Dis Clin NA 2016;30:1023–1052. 10.1016/j.idc.2016.07.008 27816138

[7] Cerulli Irelli E , Morano A , Di Bonaventura C. Reduction in nosocomial infections during the COVID-19 era: a lesson to be learned. *Updates Surg* 2020. doi: 10.1007/s13304-020-00925-0. PMC7676752 33215337

